# Pharmacokinetic/Pharmacodynamic Determination of Systemic MIC Breakpoints for Intermittent, Extended, and Continuous Infusion Dosage Regimens of Mecillinam

**DOI:** 10.1128/spectrum.03441-22

**Published:** 2023-01-30

**Authors:** Vasiliki Koumaki, Aristides Dokoumetzidis, Maria Galini Faidra Angelerou, Stavroula Baka, Indran Balakrishnan, Athanasios Tsakris

**Affiliations:** a Department of Microbiology, Medical School, University of Athens, Athens, Greece; b Department of Microbiology, Aretaieion Hospital, University of Athens, Athens, Greece; c Department of Pharmacy, University of Athens, Athens, Greece; d Department of Medical Microbiology, Royal Free London NHS Foundation Trust, London, United Kingdom; Pontificia Universidade Catolica do Parana

**Keywords:** *Enterobacterales*, mecillinam, intermittent infusion, continuous infusion, Monte Carlo simulation, pharmacokinetic model

## Abstract

Intravenous mecillinam has been used for the treatment of urosepsis at several dosing regimens, including a dose of 1,000 mg three times a day (TID). In the current pharmacokinetic/pharmacodynamic (PK/PD) study, we analyzed intermittent, extended, and continuous infusion regimens of mecillinam to provide dosage recommendations to treat infections caused by *Enterobacterales* exhibiting relatively higher mecillinam MICs than the wild-type strains. Monte Carlo simulation studies indicated that regimens of 1,000 mg TID and 1,000 to 1,200 mg four times a day (QID) are efficacious against wild-type and extended-spectrum β-lactamase-producing *Enterobacterales*, respectively. Prolonged infusion regimens (extended and continuous) could cover carbapenemase producers with a higher range of MICs (2 to 8 mg/L).

**IMPORTANCE** Previous studies have shown that intravenous mecillinam might be suitable for treatment of urosepsis. Since multidrug-resistant *Enterobacterales* are common pathogens in such infections, an effort was made to delineate intermittent, extended, and continuous infusion regimens that could cover pathogens exhibiting relatively higher mecillinam MICs than the wild-type strains. Our PK/PD analysis has shown that mecillinam might be considered a valuable therapeutic option for the treatment of systemic infections caused by extended-spectrum β-lactamase- and carbapenemase-producing *Enterobacterales* exhibiting mecillinam MICs up to 8 mg/L.

## OBSERVATION

Amdinocillin is a narrow-spectrum penicillin from the 6-amidinopenicillin group with specific and high activity against Escherichia coli and other *Enterobacterales* (1). It has been used since the early 1980s for the treatment of lower urinary tract infections, primarily in the Nordic countries (1, 2). Particularly, the intravenous (i.v.) mecillinam has been recommended by Danish guidelines for the therapy of suspected urosepsis at a dose of 1,000 mg three times a day (TID) (3). Published clinical data have also supported the use of i.v. mecillinam formulation for the cure of pyelonephritis, even in bacteremic patients, and therefore, several dosage regimens have been described (4). Nevertheless, to date, there are no official breakpoints (CLSI and EUCAST) for the treatment of systemic infections with mecillinam, except susceptibility breakpoints of pivmecillinam for uncomplicated urinary tract infections (UTIs) at MICs of ≤8 mg/L issued by EUCAST. Recent data have shown that multidrug-resistant *Enterobacterales*, including extended-spectrum β-lactamase (ESBL) producers and some carbapenem-resistant pathogens, exhibit relatively low mecillinam MICs (1 to 2 mg/L for ESBL and <8 mg/L for NDM, OXA-48, and IMP producers), albeit higher than in the wild-type population (5–7).

In the current pharmacokinetic/pharmacodynamic (PK/PD) work, we aimed to study by simulation several dosages of mecillinam, by intermittent, extended, and continuous infusion, in order to determine the regimen that would be required to treat infections caused by wild-type *Enterobacterales* (MIC range, 0.125 to 0.5 mg/L) as well as ESBL- or carbapenemase-producing strains with relatively high mecillinam MICs compared to the wild-type population.

### Population pharmacokinetic model.

Literature data previously published by Gambertoglio et al. (8), for mecillinam concentrations after i.v. administration of a single dose of 10 mg/kg of body weight to 12 healthy volunteers, as a 15-min i.v. infusion, were retrieved. We reanalyzed these data using compartmental analysis by a methodology able to retrieve both the population means for the pharmacokinetic (PK) parameters and their interindividual variabilities (IIVs) by making usage of published aggregate data of concentration mean and standard deviation values (9). The PK parameters and their variabilities were estimated as follows: using a compartmental PK model, at each estimation step, a large number of virtual patients with different parameter values, which were drawn from lognormal distributions with known mean and standard deviation, were simulated. Also, at each time point the mean and standard deviation for the simulated profiles were calculated and these two sets of values were compared (fitted) to the respective, observed, aggregate data values and the literature mean and standard deviation values. Based on that, a Markov-chain Monte Carlo estimation algorithm as implemented in Stan (https://mc-stan.org/) was used to estimate the mean and standard deviation of the compartmental PK model parameters, i.e., the population means and IIV values. The entire procedure was implemented in R version 3.5.1 (R Core Team, https://www.R-project.org/) and more specifically in RStan version 2.19.2 (https://mc-stan.org/users/interfaces/rstan), which is an R-based interface for Stan.

### Monte Carlo simulations.

Using the derived population model, Monte Carlo simulations were carried out in order to study the probability of target attainment (PTA) of various dosage regimens. The regimens studied were 1,000 mg TID, 1,000 mg four times a day (QID), and 1,200 mg QID, as previously published by Ode et al. (10), as intermittent as well as extended (prolonged intermittent) infusions over 2 and 4 h. A range of dosages (150 to 4,800 mg/day) was also administered by continuous infusion. The rationale for extended and continuous infusion was to achieve therapeutic levels for longer times given the short half-life of the drug (8). The PK profiles of 10,000 subjects (5,000 females and 5,000 males) were simulated for 5 days. Since the model was parameterized per kilogram, weight data for adult female and male populations were used as reported by the U.S. National Center for Health Statistics (11). A lognormal distribution was fitted to the percentile data reported and provided mean and standard deviation (SD) values that were used for the Monte Carlo simulations. Infusion duration was taken as 20 min. MICs studied ranged from 0.125 to 16 mg/L. Protein binding was assumed to be 10% (12). Target attainment (TA) was deemed to have been achieved when the fraction of dosing interval during which the antibiotic concentration remained above the MIC (%*f*T_>MIC_) was ≥40%, while for continuous dosage regimens, TA was achieved for practically (%*f*T_>MIC_) 100%, as previously described (13). The dosage regimen was deemed efficacious when the PTA, i.e., the proportion of patients achieving the TA, was ≥90%, which is a threshold corresponding to a reasonably high proportion of the population often used in such analyses (14). Monte Carlo simulations were carried out in the clinical trial simulation software Simulx version 2021R1 (Lixoft, https://lixoft.com/products/simulx/), and further data analysis was carried out in R. It should be noted that the entire analysis and simulations were based on the assumption that mecillinam follows linear PKs. Indeed, there is no evidence of nonlinear kinetics in literature, and with an i.v. route of administration, limited protein binding, and limited metabolism, established for mecillinam, nonlinear kinetics is unlikely. Furthermore, in the SmPC of pivmecillinam (12), a prodrug of mecillinam intended for oral administration, it is stated that “mecillinam displays linear PK in the clinically relevant range.”

### The two-compartment PK model.

**FIG 1:**
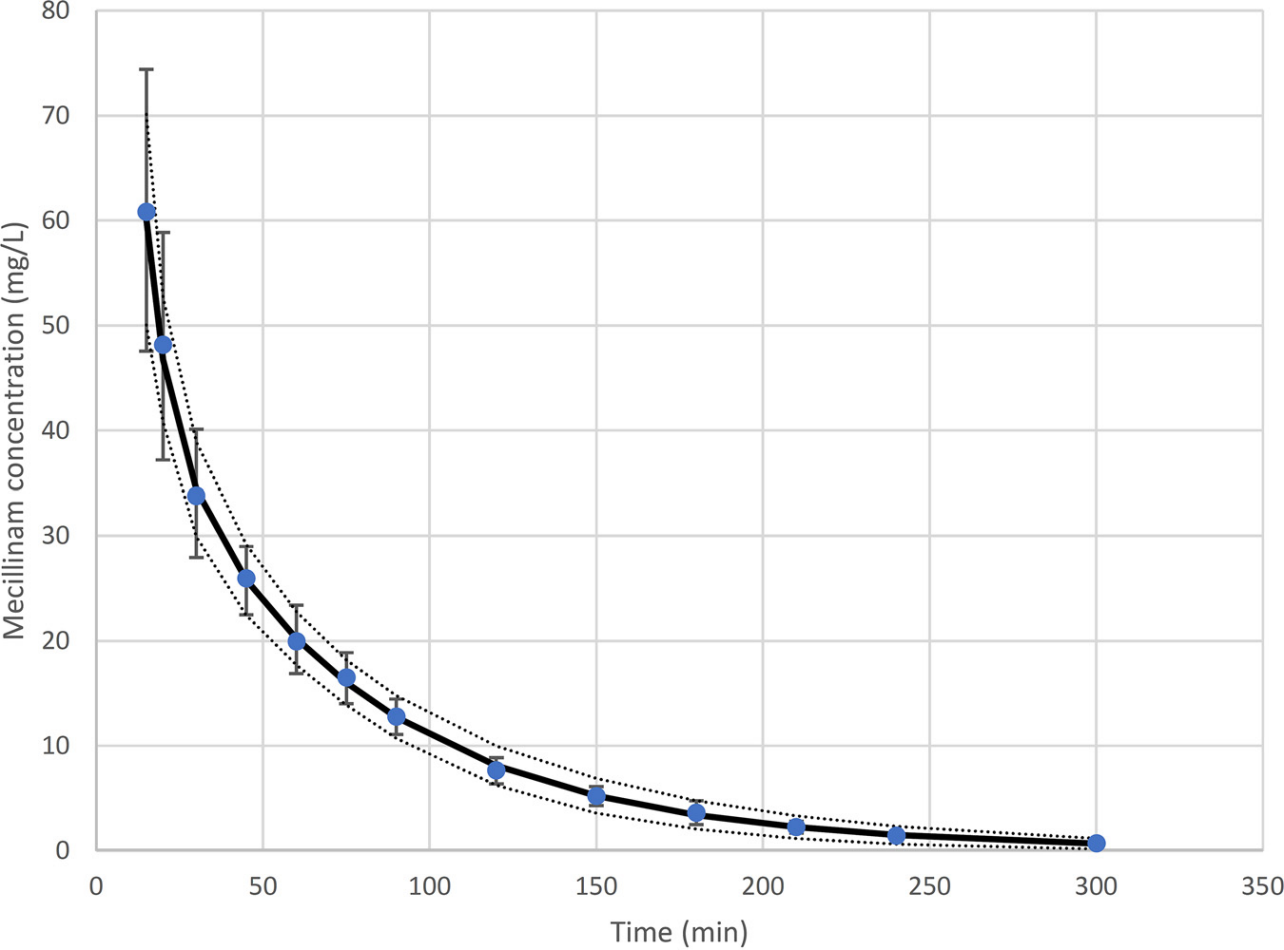
A plot equivalent to a visual predictive check. The black line corresponds to the means of the concentration predictions, and the dotted lines correspond to the mean + SD and the mean − SD, respectively, from 1,000 simulated profiles with the model, while the blue dots and the error bars correspond to the mean and SD values reported by Gambertoglio et al. (8).

Using the methodology described above, a two-compartment PK model was found to describe the data best. The PK parameter values together with corresponding IIVs of the two-compartment model were found to be as follows: clearance (CL), 3.45 mL/min/kg with IIV of 10.2%; central volume of distribution (*V*_1_), 123.12 mL/kg with IIV of 27.8%; intercompartmental clearance (*Q*), 6.74 mL/min/kg where no IIV was estimated; and peripheral volume of distribution (*V*_2_), 80.56 mL/kg with IIV of 36.0%. These values together with the corresponding standard errors, which represent the uncertainty of the estimates, are shown in Table 1. The values agree with those reported previously (8) as calculated by a noncompartmental method from the raw data, i.e., clearance of 3.5 mL/min/kg in reference 8 versus 3.45 mL/min/kg here and volume of distribution at steady state (*V*_SS_) of 230 mL/kg in reference 8 versus 202 mL/kg here, which can be calculated as the sum of *V*_1_ + *V*_2_. Furthermore, in order to assess goodness of fit, a plot within the rationale of a visual predictive check was created, by simulating 1,000 virtual patients with the model and calculating the mean and SD values of the concentrations at each time point, which were then coplotted with the observed means and SDs of the work of Gambertoglio et al. (8) (Fig. 1). It can be observed from Fig. 1 that the model predictions capture the observed data including the IIV, as observed by the corresponding SD values.

**TABLE 1:** Population pharmacokinetic parameters for mecillinam

Parameter	Population value	RSE^*a*^ (%)	Interindividual variability (%)	RSE (%)
CL (mL/min/kg)	3.45	1.8	10.2	17.1
*V*_1_ (mL/kg)	123.12	16.1	27.8	35.6
*Q* (mL/min/kg)	6.74	43.4		
*V*_2_ (mL/kg)	80.56	21.1	36.0	35.6

a RSE, relative standard error.

Monte Carlo simulation findings have shown that for the dosage regimens of 1,000 mg TID, 1,000 mg QID, and 1,200 mg QID and PTA as described above, the calculated MIC breakpoint is approximately 1 mg/L for a dosage regimen of 1,000 mg TID, and at least 2 mg/L for those of 1,000 mg QID and 1,200 mg QID. The corresponding PTAs for these dosage regimens and MICs were 90.39%, 96.55%, and 96.14%, respectively (Table 2). For extended infusion regimens over 2 h, 1,000 mg TID could allow for an MIC breakpoint of at least 2 mg/L, whereas 1,000 and 1,200mg QID could allow for a breakpoint of 4 mg/L (corresponding PTAs, 98.97%, 99.92%, and 99.94%, respectively) (Table 2). For extended infusion regimens over 4 h, 1,000 mg TID and 1,000 QID could allow for an MIC breakpoint of at least 4 mg/L, whereas 1,200 mg QID could allow for a breakpoint of 8 mg/L (corresponding PTAs, 99.88%, 100%, and 97.25%, respectively) (Table 2). For continuous infusion regimens, a dosage of 2,000 mg/day (1.4 mg/min) could allow an MIC breakpoint of at least 2 mg/L, 3,500 mg/day (2.4 mg/min) could allow an MIC breakpoint of at least 4 mg/L, and 4,800 mg/day (3.3 mg/min) could allow for an MIC breakpoint of 8 mg/L (corresponding PTAs, 99.9%, 99.6%, and 98.5%, respectively) (Table 3).

**TABLE 2:** Probability of target attainment (PTA) for 3 dosage regimens with intermittent and prolonged infusion (2 h and 4 h) for a range of MICs

MIC (mg/L)	PTA (%)^*a*^								
	Intermittent infusion, 20 min			Prolonged infusion					
				2 h			4 h		
	1 g × 3	1 g × 4	1.2 g × 4	1 g × 3	1 g × 4	1.2 g × 4	1 g × 3	1 g × 4	1.2 g × 4
0.125	**99.94**	**100**	**100**	**100**	**100**	**100**	**100**	**100**	**100**
0.25	**99.66**	**100**	**100**	**100**	**100**	**100**	**100**	**100**	**100**
0.5	**98.01**	**99.98**	**99.98**	**100**	**100**	**100**	**100**	**100**	**100**
1	**90.39**	**99.75**	**99.82**	**99.97**	**100**	**100**	**100**	**100**	**100**
2	63.99	**96.55**	**97.14**	**98.97**	**100**	**100**	**100**	**100**	**100**
4	17.93	72.38	77.09	80.72	**99.92**	**99.94**	**99.88**	**100**	**100**
8	0.46	14.37	22.29	12.9	75.27	80.82	64.98	88.26	**97.25**
16	0	0.13	0.57	0.05	4.75	11.49	2.2	9.81	25.61

a Values in boldface indicate PTAs of >90%.

**TABLE 3:** Probability of target attainment (PTA) for 6 regimens with continuous infusion for a range of MICs

MIC (mg/L)	PTA (%)^*a*^					
	150 mg/day	500 mg/day	1,000 mg/day	2,000 mg/day	3,500 mg/day	4,800 mg/day
1.125	**100**	**100**	**100**	**100**	**100**	**100**
0.25	**98.53**	**100**	**100**	**100**	**100**	**100**
0.5	33.93	**99.91**	**100**	**100**	**100**	**100**
1	0.29	64.81	**99.91**	**100**	**100**	**100**
2	0	2.17	64.74	**99.91**	**100**	**100**
4	0	0	2.16	64.81	**99.62**	**100**
8	0	0	0	2.17	50.17	**98.53**
16	0	0	0	0	0.9	33.93

a Values in boldface indicate PTAs of >90%.

Previous studies have presented PK parameters and excretion characteristics of mecillinam after i.v. administration in healthy subjects (4, 8, 15). According to their findings, mecillinam has a relatively short elimination half-life, a small volume of distribution, and a low clearance from plasma. However, population PK analyses and Monte Carlo simulation studies, which would permit mecillinam i.v. dosage individualization for the patient, have not appeared yet. In the current PK/PD study, simulation results have shown that 1,000 mg TID could cover efficiently the wild-type population of *Enterobacterales*. A dosage of 1,000 to 1,200 mg QID might also achieve adequate concentrations to treat ESBL-producing *Enterobacterales*, while the same doses given as extended infusions over 2 and 4 h, as well as the continuous infusion regimens of 2,000 mg/day, 3,500 mg/day, and 4,800 mg/day could cover *Enterobacterales* with the even higher MICs (2 to 8 mg/L) that might be seen among carbapenemase producers (4–6, 16). These data might be useful for the application of CLSI and EUCAST susceptibility breakpoints. Notably, the previously described inoculum effect with mecillinam and other β-lactams against ESBL producers, which can be reversed with the addition of clavulanate, should be taken into account (17). Therefore, combination therapies of mecillinam regimens might be considered in treating serious infections (18).

In conclusion, mecillinam might be considered a valuable therapeutic option for the treatment of systemic infections caused by *Enterobacterales*, possibly including strains that are multidrug resistant. Here, we have suggested suitable breakpoints for various regimens of mecillinam, by intermittent, extended, and continuous infusion, determined by Monte Carlo simulation. Clinical data are also needed to support these PK findings.
